# Editorial: Non-coding genome and endocrinology: from bench to bedside

**DOI:** 10.3389/fendo.2023.1264302

**Published:** 2023-09-26

**Authors:** Radu Pirlog, Kinga Nemeth, George Adrian Calin

**Affiliations:** ^1^ Research Center for Functional Genomics Biomedicine and Translational Medicine, Iuliu Hatieganu University of Medicine and Pharmacy, Cluj-Napoca, Romania; ^2^ Department of Translational Molecular Pathology, The University of Texas MD Anderson Cancer Center, Houston, TX, United States; ^3^ Center for RNA Interference and Non-Coding RNAs, The University of Texas MD Anderson Cancer Center, Houston, TX, United States

**Keywords:** non-coding RNA, endocrinologic diseases, microRNA, lncRNA, circRNA

Non-coding RNAs (ncRNAs) have emerged as crucial players in cellular processes and disease pathogenesis. In the context of endocrine diseases, the significance of these enigmatic elements is becoming increasingly apparent. The ability of ncRNAs to serve as potential biomarkers and therapeutic targets has sparked immense interest, offering new avenues for diagnostic and therapeutic interventions (1). Circulating RNAs for example, were proposed as candidates as minimally invasive biomarkers for disease progression and treatment monitoring (2). Also, several ncRNAs have been found dysregulated and indicated as potential biomarkers in diabetes mellitus, Cushing syndrome, thyroid carcinoma, pituitary adenoma, and pheochromocytoma (3–5). Understanding the roles of ncRNAs in endocrine diseases is an emerging field, which has the potential to uncover new insights into the pathophysiology of these diseases and potentially lead to the development of novel treatments.

This Research Topic aimed to gather the latest research, encompassing diverse methodologies and perspectives, to explore the multifaceted roles of ncRNAs in endocrine diseases. The Research Topic managed to bring together a wide range of topics that aim to unravel the intricate roles of ncRNAs in endocrine diseases, providing valuable insights into disease mechanisms, identification of biomarkers, and potential therapeutic avenues.

Fan et al. conducted a comprehensive overview that presents an up-to-date assessment of the current and emerging roles of circular RNAs (circRNAs) in diabetes mellitus and its associated complications. CircRNAs are a type of ncRNAs that form a covalently closed loop, which makes them more stable and resistant to degradation than linear RNAs. The authors elucidated the specific functions of circRNAs in different types of diabetes, including type 1, type 2, and gestational diabetes mellitus. Furthermore, the study also investigated the potential of circRNAs as diagnostic biomarkers and therapeutic targets for diabetes and its complications, such as diabetic retinopathy, nephropathy, and neuropathy. Due to their stability and abundance in diverse body fluids, including blood, urine, and saliva, circRNAs can be conveniently detected using sensitive and specific techniques like quantitative real-time PCR and RNA sequencing. In light of these findings, the authors propose that circRNAs could be used as non-invasive and cost-effective tools for early diagnosis, prognosis, and monitoring of diabetes and its complications.

Pan et al. provided an excellent review examining the emerging roles and mechanisms of exosomal ncRNAs (EXs-ncRNAs) in the bidirectional regulation between adipose tissue and other tissues in obesity and associated diseases. EXs-ncRNAs are ncRNAs that exit the cell in endogenous vesicles and are further swallowed through various receptors and used as signal transducer molecules. The authors showed that the expression of various ncRNAs such as microRNAs (miRNAs), long-noncoding RNAs (lncRNAs), and circRNAs are dysregulated in obesity, are involved in adipogenic differentiation, and are related to the development of metabolic syndrome and various cancers.

In the next article of the Research Topic, Zhang et al. presented an insightful analysis of the roles and applications of PIWI-interacting RNAs (piRNAs), a novel class of non-coding RNAs (ncRNAs), in urological tumors. The article focuses on elucidating the impact of piRNA dysregulation on tumor physiology and explores their potential for screening, diagnosis, and therapeutic advancements. The study presents the main roles of piRNAs, including their role in gene regulation, epigenetic modification, and transposon silencing. It discusses how these functions are relevant to cancer research, particularly in urological tumors such as prostate, bladder, and kidney cancer. The authors highlighted how piRNA expression profiles can be used to identify specific subtypes of urological tumors, and how this information can be used to guide treatment decisions. The authors also discussed the challenges and limitations of using piRNAs as a diagnostic tool and suggested future directions for research in this area.

In the last article, Xiao et al. explored the changes in the small noncoding RNAome during the senescence of human bone marrow mesenchymal stem cells (BMSCs) *in vitro*. They showed that during BMSC expansion and induction of senescence, the expression profile of various types of small ncRNAs, including miRNAs, piRNAs, and small nucleolar RNAs is altered.

Among the ncRNAs, multiple upregulated and downregulated miRNAs were revealed in passage 10 in hBMSCs. Notably, certain miRNAs with altered expression levels during subculture passages have been implicated in inhibiting osteogenesis and promoting cellular senescence. For example, miR-204, highly upregulated in passage 10 hBMSCs, has been found to regulate senescence-associated secretory phenotype (SASP) factors such as IL-6 and MMP3 and can hinder the osteogenic differentiation of mesenchymal stem cells (MSCs). Another miRNA, miR-183, was observed to increase with senescence and can inhibit BMSCs differentiation. However, the functions of the remaining differentially expressed miRNAs in senescent hBMSCs necessitate further investigation. A recent study revealed the upregulation of piRNA-36741 during hBMSC osteogenic differentiation, showing promise in mitigating osteoporosis (6). However, the association between piRNAs and BMSC senescence remains unclear, although Xiao et al. found 24 piRNAs were upregulated in senescent BMSCs. The authors managed to sequence multiple snRNAs and even to identify expression differences between transcripts, but due to limited data availability of supportive research the validation of these transcripts could not be verified. The identification of differentially expressed ncRNAs and their target genes offer opportunities for developing novel interventions to enhance the therapeutic potential of BMSCs.

This Research Topic covers a wide range of topics centered around the dysregulation of diverse families of ncRNAs and their involvement in the pathogenesis of endocrinological diseases and associated disorders. The curated manuscripts offer a new perspective on the pathogenesis of known diseases including diabetes, obesity, and metabolic syndrome. These articles specifically shed light on the emerging species of ncRNAs that have gained significant attention in recent years. Overall, the chosen articles within this Research Topic successfully captivate the interest of both clinicians and researchers and can stimulate the advancement of new translational approaches These approaches hold the potential to unravel previously unexplored mechanisms and vulnerabilities associated with endocrinological diseases, fostering the development of innovative strategies for improved diagnosis, treatment, and management.

## Author contributions

RP: Writing – original draft. KN: Writing – original draft, Writing – review & editing. GC: Supervision, Writing – review & editing.
